# Mycotoxins and plant diseases in a changing climate: from pathogen ecology to smart surveillance and mitigation strategies

**DOI:** 10.3389/ffunb.2026.1784634

**Published:** 2026-03-24

**Authors:** Ilham Dehbi, Abdelaaziz Farhaoui, Khadija Benamar, Fatima Ezzahrae Smouni, Hakima Achetoui, Mohammed Kouighat, Mouna Janati, Essaid Ait Barka, Hamid Mazouz, Rachid Lahlali

**Affiliations:** 1 Phytopathology Unit, Department of Plant and Environment Protection, Ecole Nationale d’Agriculture de Meknes, Meknes, Morocco; 2 Laboratory of Biotechnology and Bioresources Valorization. Moulay Ismail University of Meknes, Meknes, Morocco; 3 Equipe de Recherche en Didactique des Sciences et Sciences de l’Education et TICE. (DSSE_TICE), Centre Régional des Métiers de l’Education et de la Formation de Fès Meknès, Meknes, Morocco; 4 Microbial Biotechnology and Bioactive Molecules Laboratory, Sciences and Technologies Faculty, Sidi Mohamed Ben Abdellah University, Fez, Morocco; 5 Laboratory of Biology and Health, Faculty of Sciences, Ibn Tofail University, Kenitra, Morocco; 6 Research Unit of Plant Breeding and Plant Genetic Resources Conservation, Regional Agricultural Research Center of Meknes, National Institute of Agricultural Research, Rabat, Morocco; 7 Unité de Recherche Résistance Induite et Bio-Protection des Plantes-EA 4707-USC INRAE1488, Université de Reims Champagne-Ardenne, Reims, France

**Keywords:** artificial intelligence, climate change, food safety, mycotoxins, plant–pathogen interactions, surveillance

## Abstract

Mycotoxins, secondary metabolites produced by pathogenic fungi such as *Fusarium*, *Aspergillus*, *Penicillium*, and *Alternaria* pose a significant threat to global food safety and agricultural productivity. These toxins often arise concurrently with plant diseases, particularly under favorable environmental conditions that enhance fungal infection and colonization. Climate change, characterized by rising temperatures, altered precipitation patterns, and increased frequency of extreme weather events, is amplifying the occurrence and severity of both plant diseases and mycotoxin contamination. This research provides a comprehensive overview of the ecological, physiological, and molecular interplay between mycotoxigenic fungi and their host plants. We investigate how climate variables impact mycotoxin biosynthesis and pathogen virulence, as well as how host responses can be compromised under abiotic stress. Advanced omics technologies, smart diagnostics, and artificial intelligence are presented as transformative tools for early detection, risk prediction, and integrated management. Emphasis is also placed on biological control strategies, resistance breeding, and postharvest innovations for mycotoxin mitigation. Finally, we discuss the importance of regulatory frameworks and region-specific surveillance systems, particularly in vulnerable agroecosystems of the Global South. Bridging plant pathology, toxicology, and climate science, this work highlights the urgent need for holistic, climate-resilient solutions to protect crop health and ensure food security in a changing world.

## 1 Introduction

The contamination of food and feed crops by mycotoxins represents a persistent and escalating threat to global food security and public health (Adeyeye et al., 2022; Akinmoladun et al., 2025). This crisis, long recognized as a significant challenge, is now being fundamentally reshaped by the unequivocal impacts of climate change (Valero Díaz et al., 2025).

Mycotoxins are not merely agricultural contaminants but a pervasive global hazard with cascading effects on food security, international trade, and public health, disproportionately affecting vulnerable populations (Adeyeye et al., 2022; Bugingo et al., 2025). The Food and Agriculture Organization (FAO) has estimated that as much as 25% of the world’s crops are affected by mycotoxins annually, resulting in colossal losses of approximately 1 billion metric tons of food and food products (Moretti et al., 2017b; Eskola et al., 2020; Goda et al., 2025). This contamination affects a wide range of major crops, including wheat, maize, peanuts, and coffee (Reddy et al., 2010). The economic impact is multifaceted, manifesting as reduced crop value, decreased animal productivity, and high costs associated with management, litigation, and human healthcare (Gbashi et al., 2018). Estimates for the U.S. and Canada vary, with some reports suggesting an annual impact of $0.5 to $1.5 billion and others reaching as high as $5 billion (Abbas, 2005). For instance, aflatoxin-related losses alone in the U.S. maize industry are estimated at $225 million per year, not including an additional $20–30 million annually in testing and mitigation costs (Ouko, 2014). Similarly, losses from deoxynivalenol (DON) in the U.S. were estimated at $655 million per year, with the majority of the losses in wheat (Res, 2015). In developing countries, the economic burden is even more acute, with aflatoxin-related losses collectively estimated at approximately USD 900 million per year across Indonesia, the Philippines, and Thailand (Moretti et al., 2017b).

A critical element of this global crisis is the divergence in its primary impact on developed versus developing countries (Gbashi et al., 2018). In developed nations with robust monitoring and enforcement systems, the burden is predominantly economic, affecting market value, trade, and producer revenue due to rejected or downgraded commodities (Gbashi et al., 2018; Goda et al., 2025). Conversely, in Low-Income Countries (LICs), the human health cost is often the most significant and difficult to quantify impact, with mycotoxin contamination leading to food shortage crises by reducing the available supply of acceptable food (Amuzie et al., 2016). On a global scale, human health is considered the most significant mycotoxin impact, with substantial losses in monetary terms and human lives (Moretti et al., 2017b; Goda et al., 2025). Mycotoxins are known to be carcinogenic, teratogenic, and mutagenic, leading to a range of adverse health effects (Omotayo et al., 2019). Aflatoxins, for example, are linked to liver damage, while ochratoxin A (OTA) is associated with kidney damage (Qing et al., 2022). Vulnerable populations, including the very young and pregnant people, are particularly at risk, as their renal and hepatic systems are still developing and some toxins can cross the placenta and the blood-brain barrier (Kadan and Aral, 2021; Singh and Kumari, 2022). Deoxynivalenol (DON) poses significant health risks, causing symptoms like vomiting, loss of appetite, diarrhea, potential cancer development, immune system impairments, neuroendocrine disruptions, genetic damage, and in severe cases, fatality, highlighting its serious implications for food safety and public health (Murtaza et al., 2024).

The relationship between mycotoxins and plant disease is not static; it is being fundamentally altered by climate change. As a primary agroecosystem factor, climate—including temperature, precipitation, and extreme events—influences every stage of the fungal life cycle, from colonization to toxin production (Zingales et al., 2022; Casu et al., 2024). The ability of mycotoxigenic fungi to respond to these changes is inducing a shift in their geographical distribution and the patterns of mycotoxin occurrence (Perrone et al., 2020; Kos et al., 2023; Casu et al., 2024). A key impact of this phenomenon is the migration of thermophilic and thermotolerant species, such as *Aspergillus flavus*, into new, previously low-risk regions (Adewunmi and Fapohunda, 2019; Casu et al., 2024). For instance, aflatoxin contamination, historically associated with tropical and subtropical climates, is now a growing concern in temperate regions of Europe, North and South America, and Asia (Kos et al., 2023; Casu et al., 2024). Rising global temperatures, with Europe warming twice as fast as the global average, are projected to make certain mycotoxins like aflatoxin (AF), DON, and ochratoxin A (OTA) more prevalent in these new areas as conditions become more favorable for the fungi that produce them (Kos et al., 2023; Casu et al., 2024).

Beyond the direct effects on pathogens, climate change also impacts the host plants themselves (Elad and Pertot, 2014). Abiotic stressors such as extreme temperatures, drought, and heavy precipitation events weaken the natural resistance of crops, making them more vulnerable to fungal infections (Oshunsanya and Nwosu, 2019). This creates a compounding effect where a more aggressive pathogen population encounters a less resilient host, leading to a heightened risk of disease outbreaks and contamination (Engering et al., 2013; Vicente-Santos et al., 2023). Different fungal genera have distinct optimal conditions: *Aspergillus* species thrive in warmer, tropical/subtropical climates, particularly *A. flavus* and *A. parasiticus*, exhibit maximal growth at 33 °C, with a favorable range of 29-35 °C. They can grow at temperatures as low as 12 °C and as high as 43 °C under suitable conditions (Holmquist et al., 1983), while *Fusarium* species prefer cooler, more temperate conditions, thriving best at temperatures ranging from 20 °C to 30 °C, but requiring high humidity (Garcia-Cela et al., 2022; Garcia et al., 2024). For instance, *Fusarium verticillioides* showed maximum growth at 30 °C with 90% humidity, producing high levels of fumonisin B1 (Rahayu et al., 2015). A causal relationship suggests that as global temperatures rise, the geographical balance will shift, favoring the expansion of *Aspergillus* into regions currently dominated by *Fusarium* (Alkhalifah et al., 2023).

For decades, mycotoxin management has been largely reactive, focusing on detecting contamination after it has occurred and then attempting to mitigate the damage through sorting, detoxification, or disposal (Karlovsky et al., 2016; Nazareth et al., 2024). However, this approach is economically inefficient, resource-intensive, and fails to address the root causes of contamination (Karim et al., 2025b). The confluence of climate change and technological innovation necessitates a fundamental shift toward proactive, predictive, and integrated strategies (Casu et al., 2024; Polano, 2024). The advent of new technologies, including satellite remote sensing, hyperspectral imaging, and artificial intelligence (AI), is enabling a new era of “smart surveillance” (Prasanna et al., 2024). These tools allow for the non-contact, real-time monitoring of vast agricultural areas to detect plant stress and disease symptoms long before they are visible to the human eye. The integration of AI and machine learning (ML) with remote sensing data is a game-changer (Aggarwal et al., 2024). These algorithms are trained on large, labeled datasets to recognize patterns associated with healthy and diseased plants, enabling the rapid classification and quantification of contamination (Ali et al., 2024). Studies demonstrate that ML algorithms, such as multilayer perceptron (MLP) and support vector regression (SVR), achieve accuracies exceeding 98% in detecting aflatoxin B1 in contaminated samples (Wang et al., 2023; Kabir et al., 2024). Beyond large-scale surveillance, the development of portable, on-site detection tools is democratizing food safety. These include smartphone-based apps that use lateral flow devices (LFDs) to provide inexpensive and rapid quantitative mycotoxin analysis in the field (Ibrahim, 2020; N.B. et al., 2024).

No single strategy can effectively combat the complex and dynamic nature of mycotoxin contamination (Nazareth et al., 2024). The most effective approach is an integrated one, combining strategies at every stage of the food chain—from pre-harvest to post-harvest. This includes the breeding of resistant cultivars, the use of biological control agents, and the implementation of sound agronomic and storage practices (Moretti et al., 2017a). A foundational approach is the breeding of disease- and stress-resistant cultivars, which, while effective, can be slow and complex (Gadag et al., 2021). Biological control is another promising intervention, involving the use of non-toxigenic fungal strains and microbial antagonists to competitively exclude toxin-producing strains (Ehrlich et al., 2014). For example, the use of atoxigenic *Aspergillus flavus* strains has shown significant success, reducing aflatoxin contamination in peanuts and cotton by 70-90% (Shabeer et al., 2022). At the post-harvest stage, proper drying of crops to safe moisture levels (typically 10-13% for cereals) and good storage management are critical preventive measures (Kharayat and Singh, 2018; Kumari et al., 2021). Sorting tests showed that aflatoxin was reduced by an average of 46% in one pass and up to 88% with a second pass, effectively demonstrating that sorting damaged and moldy kernels is a highly effective method for mycotoxin reduction (T. C. Pearson et al., 2009). In contrast, biological methods, such as the use of mycotoxin-degrading microbes, are described as more efficient, specialized, and ecologically friendly (Oztekin et al., 2023; Wang et al., 2024). The interconnected nature of mycotoxin contamination—linking pathogen ecology, crop health, animal feed, human food, and environmental factors—mandates a “One Health” approach (Ogunseitan, 2022). This framework recognizes the complex and symbiotic relationships between human, animal, and environmental health, arguing that a solution for one must consider its impact on the others. A truly effective solution requires moving beyond disciplinary silos, necessitating integrated and interdisciplinary approaches involving agronomists, mycologists, climate scientists, post-harvest handlers and processing experts, medical researchers, and policymakers.

Significant challenges remain in closing knowledge gaps and translating research into practice, including the need for more complex predictive models and effective technology transfer to resource-limited settings. Mycotoxins and plant diseases represent a critical and growing threat, amplified by a changing climate. The solution lies in a profound paradigm shift: from a reactive stance of post-contamination cleanup to a proactive, predictive, and integrated management system. This review synthesizes cutting-edge research to present a holistic, multi-faceted understanding of this crisis, moving beyond traditional reactive paradigms toward a proactive, integrated management framework.

## 2 Mycotoxigenic fungi

Mycotoxins are secondary metabolites produced by various fungi. Although they differ in structure and biosynthesis pathways, all known mycotoxins are chemically stable and can survive most food processing stages, especially in animal feed, leading to contamination of the final product (Casu et al., 2024). Fungal growth in produce and stored products causes losses in both yield and quality, resulting in significant monetary damage (Adeyeye, 2016). Although the mycotoxin issue has persisted for decades, the development of more sensitive analytical tools and stricter regulations in many countries has greatly increased awareness and urgency surrounding this problem (Logrieco et al., 2018). This has created a positive feedback loop where improved detection capabilities lead to more reported cases, which in turn boosts demand for tighter controls and more research into effective solutions (Szelenberger et al., 2024).

This review section emphasizes the four significant mycotoxigenic genera of fungi: *Aspergillus*, *Fusarium*, *Penicillium*, and *Alternaria*. The argument here is to provide a comprehensive understanding of their specific ecological niches, the typical toxins they exhibit, the crop commodities they tend to infect, and the documented health effects of such toxins. The report shall also ponder the complex issues of mycotoxin co-occurrence and the multifaceted strategy for effective mitigation. To be a helpful and brief reference, **Table 1** below briefly lists the principal genera, their principal toxins, and the most frequently involved commodities in contamination.

**Table 1:** Major mycotoxigenic fungi, their principal toxins, and associated food commodities.

Genus	Key species	Major toxins	Common commodities	References
*Aspergillus*	*A. flavus*, *A. parasiticus*, *A. niger*, *A. clavatus*	Aflatoxins (AFs), Ochratoxin A (OTA), Patulin (PAT)	Maize, peanuts, spices, pistachios, coffee, rice, wheat, dried fruits	(Adeyeye, 2016; Perrone and Gallo, 2017; Abarca et al., 2019; Winter and Pereg, 2019; Pickova et al., 2021)
*Fusarium*	*F. graminearum*, *F. verticillioides*, *F.* sp*orotrichioides* *Fusarium* sp.	Fumonisins (FUMs), Trichothecenes (DON, T-2), Zearalenone (ZEN), Enniatin B	Cereals: Maize, wheat, barley, rice, rye, oat, millet	(Reddy et al., 2009; Matić et al., 2013; Prosperini et al., 2017; Wang et al., 2019; Rechcígl and Rechcígl, 2020; Shabeer et al., 2021; Ekwomadu and Mwanza, 2023)
*Penicillium*	*P. expansum*, *P. citrinum*	Patulin (PAT), Ochratoxin A (OTA), and Citrinin	Apples, pears, apple juice, cereals, coffee, dried fruits	(Puel et al., 2010; Assaf, 2018; Costa et al., 2019; Gupta, 2025)
*Alternaria*	*A. alternata*, *A. tenuissima*	Alternariol (AOH), Tenuazonic acid (TeA), Alternariol monomethyl ether (AME)	Wheat, sorghum, barley, sunflower seeds, tomatoes, fruits, and vegetables	(Scott, 2001; Ostry, 2008; Alexander et al., 2011; Adeyeye, 2016; Pinto and Andrea., 2017)
	*Alternaria alternata*	Altertoxin II	Rice	(Del Favero et al., 2020)
	*Alternaria alternata*	Tentoxin	Dicotyledonous plants: soyban, corn	(Lax et al., 1988)

### 2.1 *Aspergillus* spp.: the ubiquitous producer of aflatoxins

The genus *Aspergillus* encompasses a diverse group of mycotoxigenic fungi that play a crucial role in food safety and agriculture (Adeyeye, 2016). These fungi can act both as field pathogens and storage contaminants, infecting crops before harvest as well as during post-harvest handling and storage (Choudhary, 2010). *Aspergillus* spp. are especially common in tropical and subtropical regions, where warm temperatures and high humidity create ideal conditions for their proliferation. Among these, the two most significant species from a mycotoxin standpoint are *A. flavus* and *A. parasiticus*, which are the main producers of aflatoxins (AFs) (Adeyeye, 2016; Perrone and Gallo, 2017). Another notable species, *A. niger*, is known for producing ochratoxin A (OTA) contamination (Abarca et al., 2019). Although Patulin (PAT) is more commonly associated with *Penicillium* species, certain *Aspergillus* spp. such as *A. clavatus* can also synthesize this toxin (Sajid et al., 2019; Bata-Vidács et al., 2024). Aflatoxins, a group of mycotoxins including AFB1, AFB2, AFG1, and AFG2, are most notably characterized by the high toxicity and frequent occurrence of Aflatoxin B1 (AFB1) (Adeyeye, 2016). These toxins are predominantly linked to tropical and subtropical crops such as maize, peanuts, cottonseed, spices, and pistachios (Winter and Pereg, 2019; Pickova et al., 2021). Contamination of these commodities with aflatoxins significantly diminishes their commercial value and makes them unsuitable for either animal feed or human consumption (Xue et al., 2025). In addition, ochratoxin A (OTA), produced by various species of *Aspergillus* and *Penicillium*, is a widespread mycotoxin that contaminates a large number of staple foods, including cereals, coffee beans, dried fruits, wine, grape juice, and spices (Barkai-Golan, 2008; Nazareth et al., 2024). Patulin (PAT), a polyketide lactone mycotoxin produced by certain species of *Aspergillus* and *Penicillium*, mainly infects fruits such as apples, peaches, and apricots (Lauren Jackson, 2005; Sajid et al., 2019).

### 2.2 *Fusarium* spp.: the cereal pathogen and its diverse array of mycotoxins

*Fusarium* is mostly fungi that dwells in fields and is rated as the globe’s most important plant pathogens and causes of many devastating infections, including wilting, scorching, and rotting, in economically valuable crops, particularly cereals such as corn and wheat (Shabeer et al., 2021; Ekwomadu and Mwanza, 2023). In contrast to *Aspergillus* and *Penicillium* spp., which infect crops during storage, *Fusarium* growth and toxin development are more likely to occur before or immediately following harvest, particularly for crops overwintering in the field (Sarrocco and Vannacci, 2018). *Fusarium* produces a complex and diverse group of mycotoxins (Munkvold, 2017). The main mycotoxigenic species include: *F. graminearum*, a major producer of trichothecenes, deoxynivalenol (DON) and nivalenol (NIV), as well as the estrogenic mycotoxin zearalenone (ZEN) (Wang et al., 2019); *F. verticillioides* and *F. fujikuroi*, the main synthesizers of Fumonisins (FUM) (Matić et al., 2013); and finally *F.* sp*orotrichioides*, the species responsible for the production of the highly toxic T-2 toxin, which was associated with Alimentary Toxic Aleukia (ATA) in the 1940s (Rechcígl and Rechcígl, 2020). These toxins are most frequently found in cereals such as corn, wheat, barley, and rice (Reddy et al., 2009; Shabeer et al., 2021; Ekwomadu and Mwanza, 2023). The understanding of species-mycotoxin associations within the *Fusarium* genus has been historically complicated by taxonomic confusion (Stakheev et al., 2016). This has been a significant challenge for risk assessment, as isolates producing the same toxin have been given different names, leading to inconsistent data and hindering the development of targeted control strategies (Stakheev et al., 2016).

### 2.3 *Penicillium* spp.: a major agent of post-harvest mycotoxin contamination

The genus *Penicillium* is primarily known as a storage fungus that grows on very moist substrates and is one of the main causes of post-harvest rot (Adeyeye, 2016). While some species of this genus, e.g., *P. notatum*, have been of utmost benefit to medicine via the production of antibiotics such as penicillin (Moss, 2002), others are major producers of mycotoxins that present a food safety hazard, e.g., species of *P. expansum*, the major source of patulin (PAT), namely in pome fruits, and also producers of the characteristic “blue pustules” and rapid soft rot described on apples and pears (Puel et al., 2010). Accordingly, PAT is an important contaminant in apple products such as juice, cider, and compotes. Like *Aspergillus*, certain *Penicillium* species, e.g., *P. citrinum*, produce ochratoxin A (OTA) and citrinin (CIT) as well (Assaf, 2018; Gupta, 2025). The mycotoxins are found in a broad range of commodities like cereals, coffee, dried fruits, and spices (Costa et al., 2019). A key characteristic of these toxins is their chemical stability; they are resistant to high temperatures, meaning they can survive common food processing methods such as pasteurization (Bento de Carvalho et al., 2024). This stability emphasizes the importance of preventing fungal growth at the source, as detoxification methods after contamination are often ineffective.

### 2.4 *Alternaria* spp.: the emerging concern and its array of toxins

*Alternaria* species are significant plant pathogens that are frequently responsible for contaminating crops in the field (Escrivá et al., 2017; Pinto and Andrea., 2017). They are often the primary contaminating fungi in cereals such as wheat, sorghum, and barley, and are also a major source of spoilage for fruits and vegetables, even under refrigerated storage conditions (Scott, 2001; Ostry, 2008; Adeyeye, 2016). *A. alternata* is the most common mycotoxin-producing species within the genus. The most frequently detected toxins are alternariol (AOH), alternariol monomethyl ether (AME), and tenuazonic acid (TeA) (Escrivá et al., 2017). Other less common toxins include altertoxins (ATX), tentoxin (TEN), and altenuene (ALT) (Pinto and Andrea., 2017). *Alternaria* toxins are classified as “emerging” mycotoxins because they are frequently detected in food but lack the extensive toxicological data and legal maximum limits of other regulated mycotoxins (Zhang et al., 2025).

## 3 Host-pathogen interaction and disease symptoms

**Figure 1:**
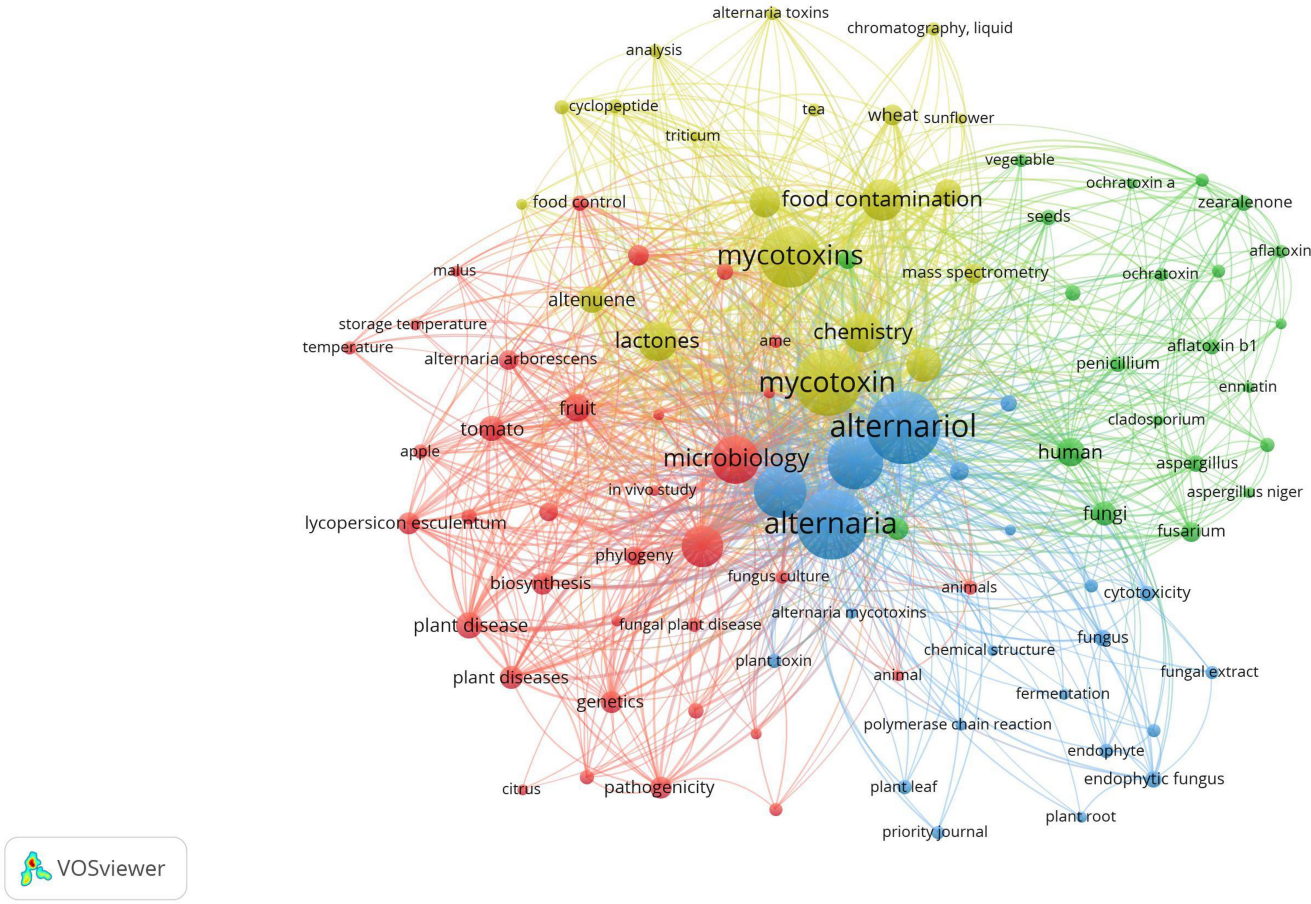
Scientific mapping of strictly linked networks for different mycotoxins and crops, based on papers identified during the research process. This map was elaborated and created using VOS viewer. (TITLE-ABS-KEY mycotoxin* OR “fungal toxin*” OR aflatoxin* OR ochratoxin* OR zearalenone OR fumonisin* OR trichothecene* OR deoxynivalenol OR DON OR “T-2 toxin”) AND TITLE-ABS-KEY (*Aspergillus* OR *Penicillium* OR *Fusarium* OR *Alternaria*) AND TITLE-ABS-KEY (changing climate)) AND PUBYEAR > 2007 AND PUBYEAR < 2025.

The relationship between mycotoxigenic fungi and their plant hosts is a dynamic interplay of pathogenicity, environmental factors, and molecular signaling. Mycotoxins are not merely inert byproducts of fungal growth but are often central to the fungus’s ability to colonize and thrive in its host. The pathogenic strategies, environmental triggers, and resulting plant symptoms vary significantly among the dominant fungal genera (**Figure 1**).

Aflatoxins are produced by *Aspergillus* species, especially *A. flavus* and *A. parasiticus*, which are known worldwide for this capability. While these fungi are commonly associated with post-harvest contamination during storage, they are also capable of infecting crops under preharvest field conditions. For example, peanut crops may become infected through direct contact with contaminated soil, whereas in maize, infection often occurs via the silks during flowering. In both cases, fungal spores frequently originate from crop residues and organic debris persisting in the soil from previous growing seasons, serving as primary inoculum sources (Shabeer et al., 2022; Gachara et al., 2024). Their pathogenic lifestyle is best described as opportunistic and facultative, as they can live as saprophytes, symptomless endophytes, or weak phytopathogens (Pfliegler et al., 2020).On key crops such as maize, peanuts, and tree nuts, *A. flavus* breaks down plant tissues to absorb nutrients, though this colonization may not always result in obvious disease symptoms on the plant itself (Klich, 2007). The primary concern with *Aspergillus* infection is not a visible disease, but the subsequent contamination of the grain with aflatoxins (Shabeer et al., 2022). A major factor influencing *A. flavus* colonization and aflatoxin production is abiotic stress, particularly drought and heat (Fountain et al., 2014). These stressors create a high degree of environmentally induced variability in host susceptibility. Rather than a random metabolic event, the production of aflatoxins appears to be a purposeful biological response to these environmental cues. Research indicates that *A. flavus* increases aflatoxin production when confronted with oxidative stress, a condition commonly encountered when colonizing plants (Klich, 2007; Fountain et al., 2014). This has led to the hypothesis that aflatoxins may function as antioxidants, scavenging free radicals to protect the fungus during its colonization process (Fountain et al., 2014; Finotti et al., 2021). In this context, the toxin serves a dual purpose: it is a tool for self-preservation that also acts as a virulence factor. Aflatoxins are phytotoxic, inhibiting plant photosynthesis by hindering chlorophyll and carotenoid synthesis, which can lead to visible symptoms like virescence or albinism in the contaminated plant (Ismaiel and Papenbrock, 2015). This dual role—as a protective agent for the fungus and a damaging agent for the host—highlights the sophisticated nature of this host-pathogen interaction.

### 3.1 *Fusarium*-mycotoxin: a true pathogen’s arsenal

In contrast to the opportunistic nature of *Aspergillus*, *Fusarium* species are renowned as destructive pathogens on cereal crops, producing mycotoxins before or immediately after harvest (Ekwomadu and Mwanza, 2023). The infection process for species like *Fusarium graminearum* typically begins with the fungus overwintering on infected crop residues and then infecting new plants through wounds or natural openings like stomata (Boenisch and Schäfer, 2011).The colonization process is aggressive and is facilitated by the production of a suite of cell-wall-degrading enzymes such as cellulases, pectinases, and xylanases, which soften and dissolve host tissues, allowing for rapid invasion (Perincherry et al., 2019). This aggressive pathogenesis leads to clear, visible disease symptoms. In wheat and barley, *F. graminearum* causes Fusarium Head Blight (FHB), characterized by the bleaching of spikelets and the presence of pink to orange masses of spores on the infected heads (Alisaac et al., 2023). Infection can also lead to the formation of shriveled, discolored, and lightweight kernels, colloquially known as “tombstone” kernels, which have poor germination rates and can result in seedling blight (Odenbach, 2009). In maize, the fungus causes stalk rot, which results in lodging, premature senescence, and yield loss due to impaired grain filling (Quesada-Ocampo et al., 2016). A key difference in the *Fusarium* model is that its mycotoxins, particularly deoxynivalenol (DON) and zearalenone (ZEA), are integral to the pathogenic process itself, functioning as essential virulence factors (Kanja et al., 2021). Studies involving mutants lacking mycotoxin production have demonstrated that although these strains can still induce disease, their virulence is markedly diminished. This evidence highlights the crucial role of toxins in overcoming host defenses, rather than simply being incidental byproducts (Quesada-Ocampo et al., 2016). For instance, fumonisins produced by *F. verticillioides* function as sphingosine analogs, hindering ceramide synthase in the host plant and thereby disrupting sphingolipid metabolic processes. Since this pathway is vital for membrane integrity and cell signaling, the fungus actively interferes with it (Zeng et al., 2020). Such interference results in cell rupture and tissue damage, representing a direct molecular mechanism of toxicity that facilitates fungal invasion and nutrient uptake (Perincherry et al., 2019). This direct link between toxin production and disease severity establishes the *Fusarium*-mycotoxin system as a prime example of mycotoxins serving as true virulence factors in plant pathology (Adam et al., 2015).

### 3.2 *Penicillium*-mycotoxin: post-harvest decay

*Penicillium* species are quintessential post-harvest fungi, typically colonizing fruits and grains during drying, storage, and processing (Greeff-Laubscher et al., 2019). Their pathogenic lifestyle is largely opportunistic, as they gain entry to the host plant via pre-existing wounds, bruises, or cracks (Dukare et al., 2022). On fruits such as apples and citrus, this infection manifests as a “blue mold” or “green mold” (Godana et al., 2023).The rot begins as a soft, circular, water-soaked lesion that spreads rapidly through the fruit’s flesh (Jurick and Adaskaveg, 2024).

### 3.3 *Alternaria*-mycotoxin: a pathogen of crops and a growing public health concern

While *Aspergillus*, *Fusarium*, and *Penicillium* are the main producers of mycotoxins, the genus *Alternaria* plays a significant role as a fungal pathogen affecting crops and is increasingly recognized as a food safety concern. With over 70 toxins identified, species such as *Alternaria alternata* are notorious for causing both pre-harvest and post-harvest diseases across various agricultural products, including cereals, oilseeds, fruits, and vegetables (Saleh et al., 2024). *Alternaria*’s pathogenic approach centers on generating host-specific toxins that are crucial for its virulence. The fungus releases diverse molecules, such as enzymes that break down cell walls and specific toxins, to infect host plants and overcome their defenses (Meena et al., 2017; Fernandes et al., 2023). For example, on apples, *A. alternata* produces AM-toxin, a cyclic peptide toxin that causes necrotic spots similar to the damage inflicted by the fungus (Wang et al., 2022). This toxin disrupts the host cell’s plasma membrane, chloroplasts, and nucleus, resulting in cell death and leaf shedding (Cao et al., 2024). In carrots, *A. dauci* causes leaf blight, which manifests as dark, irregular necrotic patches on the leaves and petioles, weakening the plant and leading to notable reductions in yield (Naqvi, 2004; Selvakumar and Kalia, 2022). From a toxicological perspective, *Alternaria* produces toxins such as alternariol (AOH) and alternariol monomethyl ether (AME), which are classified as “emerging mycotoxins” due to the lack of established regulatory limits in food products despite their frequent presence in widely consumed foods (Aichinger et al., 2021). Human exposure mainly occurs through diet, and these toxins have been linked to adverse effects in animals, including fetal toxicity and developmental abnormalities (Awuchi et al., 2021). In humans, their mutagenic and genotoxic potential observed in laboratory studies raises health concerns (Louro et al., 2024). There are also indications of a possible association between *Alternaria* toxins in grains and esophageal cancer in certain Chinese regions (Ji et al., 2023). Additionally, the structural resemblance of AOH to estrogen has prompted worries about possible endocrine-disrupting effects (Thenuwara et al., 2024). Considering the risks associated with long-term dietary exposure, there is a pressing need for more detailed toxicological research on these mycotoxins. **Table 2** provides a comparative analysis of the distinct pathogenic strategies employed by these three genera, highlighting the unique roles their mycotoxins play in their respective host-pathogen interactions.

**Table 2:** Pathogenic mechanisms of mycotoxins in host-fungus interactions.

Fungal genus	Ecological niche	Key mycotoxin(s)	Role of toxin in pathogenesis	Specific disease symptoms on plant hosts	References
***Aspergillus***	Facultative pathogen, storage fungus	Aflatoxins, OTA	Antioxidant, stress response, phytotoxicity, competition	Virescence/albinism in some plants; general decay/mold	(Razzaghi-Abyaneh et al., 2014; Ismaiel and Papenbrock, 2015)
***Fusarium***	Destructive field pathogen	DON, ZEA, Fumonisins	Essential virulence factor; host cell wall degradation; disruption of host metabolism	Fusarium Head Blight (FHB), “tombstone kernels,” stalk rot	(Odenbach, 2009; Kanja et al., 2021; Alisaac et al., 2023)
***Penicillium***	Post-harvest storage fungus	Patulin, OTA	Facilitates opportunistic infection in wounded hosts; competition	Blue/Green mold, soft rot in fruits	(Greeff-Laubscher et al., 2019; Dukare et al., 2022; Godana et al., 2023; Jurick and Adaskaveg, 2024)
***Alternaria***	Pathogen of crops	Alternaria toxins (e.g., AOH, AME, AM-toxin)	Essential virulence factor; induces cell death, membrane, and chloroplast damage	Leaf spots, necrotic lesions, defoliation, soft rot	(Meena et al., 2017; Aichinger et al., 2021; Cao et al., 2024; Saleh et al., 2024)

## 4 Dynamics of mycotoxin contamination in the agri-food chain

The contamination of agricultural commodities is not a single event but rather a continuous process that may occur at different points along the food and feed supply chain (Manning et al., 2005). It usually starts in the field, during the pre-harvest stage, when toxigenic fungi infect susceptible crops (Awuchi et al., 2021). If not controlled, contamination can persist and even worsen during post-harvest stages such as drying, storage, and transportation (Osei-Kwarteng et al., 2024).

During the preharvest stage, the interaction between crops, fungal pathogens, and environmental conditions has a significant impact on the level of contamination (Nada et al., 2022; Bereziartua et al., 2025). Climate change is reshaping this interaction by altering rainfall distribution, increasing average temperatures, and intensifying extreme events, such as droughts and heatwaves (Kos et al., 2023). These shifts create new ecological windows that enable mycotoxigenic fungi to expand into previously unsuitable areas (Casu et al., 2024). For instance, *Aspergillus* species, traditionally confined to warmer latitudes, have recently been reported in temperate regions of Southern and Eastern Europe, coinciding with a higher incidence of aflatoxin outbreaks (Pickova et al., 2021; Kos et al., 2023). Alternating patterns of rainfall and high temperatures lead to extended periods of humidity in maize and wheat fields, creating favorable conditions for fungal growth (Zingales et al., 2022; Kos et al., 2023). On the other hand, prolonged drought conditions weaken the plants’ defenses, making them more vulnerable to invasion by aflatoxin-producing fungi (Nazareth et al., 2024). A case study from northern Italy illustrates this complexity, during a season with consistently high temperatures of around 30 °C, maize fields saw a 400-fold increase in *Aspergillus* colonization and a 250-fold rise in aflatoxin contamination compared to a cooler year (Leggieri et al., 2020). Cooler and wetter conditions led to an increase in *Fusarium* species, indicating that climate stress not only heightens contamination but also alters the competitive balance among fungal populations (Kos et al., 2023; Casu et al., 2024). These findings indicate that preharvest contamination is influenced more by a complex interaction of climate, host susceptibility, and microbial competition than by the presence of a single pathogen (Bartholomew et al., 2021). Therefore, effective management strategies should adopt flexible approaches that address multiple pathogens simultaneously rather than relying solely on narrow, pathogen-specific control measures.

Although harvest marks the end of field exposure, it does not eliminate the risk of mycotoxin accumulation (Logrieco et al., 2021). Once grain is collected, the critical factors determining contamination are moisture and temperature during handling and storage if drying is delayed or inadequate, fungi established in the field may continue to grow, sometimes at an accelerated rate (Kulazhanov et al., 2024). Rapid drying to safe moisture levels (<13%) is therefore considered the most effective first-line defense, although it cannot reduce toxins already present (Choudhary, 2010). Storage facilities also play a decisive role: poorly ventilated or dirty silos, combined with temperature fluctuations, create microclimates conducive to fungal growth, and condensation on silo walls can lead to secondary contamination of stored grain. It is therefore very important to regularly monitor the temperature and humidity of grain (Zheng et al., 2024). Furthermore, mechanical injuries that occur during harvesting, transportation, or processing increase the risk of contamination by exposing nutrient-rich tissues, which are more susceptible to fungal colonization (Leyva Salas et al., 2017). Although sorting and removal of damaged kernels can reduce contamination, these practices are often costly and not always feasible at a large scale (Karlovsky et al., 2016). From a systems perspective, the postharvest stage can be seen as a hierarchy of defenses (Szelenberger et al., 2024). Preventive measures—such as drying, proper cleaning of equipment, and hygienic storage—form the foundation (Matumba et al., 2021). If these fail, producers must resort to corrective measures such as chemical detoxification or the use of mycotoxin binders, which are generally less efficient and more expensive. This highlights the importance of early intervention, since failures at the initial stages of postharvest management are difficult to compensate later (**Table 3**).

**Table 3:** Critical control points and mitigation strategies in mycotoxin management.

Contamination stage	Critical control points	Mitigation strategies	References
Preharvest	Climate Stress, Pest/Disease Pressure, Soil Conditions	Breeding resistant varieties, Crop rotation, Tillage, Pest management	(Focardi, 2022)
Harvest	Timeliness of harvest, Physical damage to kernels	Timely harvesting at optimal maturity, Proper combine settings to minimize damage	(Alt et al., 2019)
Postharvest Storage	Kernel moisture, Temperature, Humidity	Rapid and proper drying, Temperature-controlled storage, Continuous moisture and temperature monitoring	(Lavkor and Var, 2017; Venslovas et al., 2022)
Processing & Distribution	Cross-contamination, Residual toxins	Rigorous equipment and facility cleaning, Physical separation of damaged grain, Mycotoxin-binding agents (adsorbents)	(Zhang et al., 2024a)

## 5 Climate change and mycotoxin dynamics

Climate change is recognized as one of the key drivers influencing mycotoxin dynamics at a global level, via changes in environmental components that regulate the development of mycotoxigenic fungi, their global distribution, and toxigenic ability (**Table 1**, **4**). Mycotoxins are toxic secondary metabolites synthesized by filamentous fungi belonging to the genera *Aspergillus*, *Fusarium*, and *Penicillium*, and their occurrence in food and feed commodities poses serious risks to animal and human health, food safety, and global trade (Assunção et al., 2018). Future changes in thermal, hydric, and atmospheric regimes— most notably, global mean temperature increase, altered precipitation patterns, increased floods and droughts, and elevated CO_2_ levels—are creating new ecological niches for the fungi, reshaping risk areas (Mshelia et al., 2020).

**Table 4:** Regional case studies on the effect of climate change on mycotoxin dynamics.

Region	Dominant mycotoxins	Climatic conditions	Observed effects	References
Southern Europe	Aflatoxin B1 (*A. flavus*, *A. parasiticus*)	Hot summers (>30 °C), prolonged droughts, and post-drought humidity	Northward expansion of risk zones, increased maize contamination in dry years.	(Battilani, 2016; Medina et al., 2017)
Northern Europe	DON, ZEN (*F. graminearum*, *F. culmorum*)	Wetter springs/summers, moderate temperatures (20–25 °C)	Increase in Fusarium head blight and higher DON levels in wheat.	(Van der Fels-Klerx et al., 2016)
Eastern Europe	Aflatoxin B1	Exceptionally hot, dry summers	2012 crisis: massive exceedance of legal limits, milk contamination with AFM1	(Camardo Leggieri et al., 2019)
East Africa	Aflatoxins	High temperatures, postharvest humidity	Human poisoning outbreaks, mortality linked to AF.	(Edgar Mugizi et al., 2021)
West Africa	Aflatoxins, fumonisins	Alternating drought and heavy rainfall	Frequent AF + FUM co-contamination, increased health risks.	(Aasa et al., 2022)
North America	Aflatoxins, fumonisins	Hotter, drier summers, water stress	Northward shift of aflatoxin risk zones, increased FUM in maize.	(Gomes et al., 2024)
South America	DON, fumonisins, aflatoxins	Warmer summers, intense rainfall during flowering	Increased DON and FUM, emergence of AF in previously unaffected areas.	(Demonte et al., 2024)
South Asia	Aflatoxins, trichothecenes	High temperatures, irregular monsoons	Higher aflatoxin risk, frequent contamination of stored products.	(Chatterjee et al., 2023)
East Asia	DON, ZEN, fumonisins	Hot, humid summers, increased precipitation	Expansion of *F. graminearum*, higher DON levels in wheat, and increased Fusarium head blight (FHB) incidence.	(Sun et al., 2017)
Australia	Aflatoxins, DON	Alternating drought and flooding	Higher risk of postharvest contamination and toxin development during storage.	(I Pitt and D Hocking, 2003)

In the case of temperate and cold climate regions, such as much of Europe, by 2050, projections indicate that the climate will be milder and wetter (Pleadin et al., 2012; Koletsi et al., 2021). This climate change favors *Fusarium graminearum* over others, such as *F. culmorum*, which are the major producers of type B trichothecenes, including deoxynivalenol (DON) and its acetylated derivatives, as well as zearalenone (ZEN) (Gab-Allah et al., 2023). Growth can occur between 4 °C, but with a maximum of 25 °C, while mycotoxin production has a maximum between 15 °C and 25 °C (Mannaa and Kim, 2017). Experiments under controlled environments have demonstrated that elevated CO_2_ (1,000 ppm as opposed to the current 400 ppm) with limited water stress (water activity of 0.95) can significantly enhance DON and ZEN biosynthesis in certain *F. asiaticum* strains, which have the potential to exceed EU legislative limits (Cendoya et al., 2018). Intraspecific variation, demonstrated through studies of three various strains (CH024b, 82, 0982), indicates that metabolic response to future climatic conditions is far from homogeneous (Ljunggren et al., 2020).

Higher temperatures and lower rainfalls during the growing season in tropical and subtropical areas will favor aflatoxin-producing species such as *A. flavus* and *A. parasiticus*, which produce aflatoxin B_1_ (AFB_1_)—the most toxic of the group (Camardo Leggieri et al., 2019). The thermotolerant strains grow optimally in 30 °C to 37 °C, with AFB_1_ production enhanced under low water activity conditions (≤ 0.93) (Kamle et al., 2019; Roucou et al., 2021). Severe contamination has already been reported in the U.S. Corn Belt in hot, dry summers and in East Africa and India during long dry spells (Mwafulirwa, 1999). It is predicted that these conditions will gradually become more prevalent, giving more likelihood of exceeding regulatory levels in food (Adewunmi and Fapohunda, 2019; Kos et al., 2023).

Fumonisins (FUM), produced mainly by *F. verticillioides* and *F. proliferatum*, are a second group of toxins that are strongly linked to agroclimatic conditions (Zingales et al., 2022). Common in maize and maize products, fumonisin production is optimum at 15 °C and 25 °C (Roucou et al., 2021). In the future, their prevalence is expected to increase in already temperate regions (Central Europe, North America) with longer hot, wet summers and alterations in flowering and preharvest intervals—intervals of the most critical infection (Kos et al., 2023; Casu et al., 2024). Recent observations in Central Europe already show rising fumonisin contamination over the past two decades (Moretti et al., 2019).

Beyond “masked” toxins, climate change also affects the formation of modified mycotoxins and emerging toxins (Casu et al., 2024). Modified mycotoxins, resulting from plant enzymatic transformations (e.g., DON-3-glucoside) or chemical changes during postharvest stages, can accumulate alongside native forms, increasing total toxic load and complicating detection (Bryła et al., 2018). Future conditions may also favor lesser-known toxin-forming species, including possible replacement of *A. flavus* by more thermophilic species like *A. fumigatus* in certain tropical areas with gliotoxin or other potentially toxic secondary metabolite production (Paterson and Lima, 2017).

The cumulative effects of increasing temperatures, elevated CO_2_, altered moisture conditions, and more frequent extreme events will likely produce years of anomalously high contamination in alternating cycles with low-pressure phases, and prediction and prevention will be made harder (Cendoya et al., 2018). Effective mycotoxin risk management in this case will entail combined strategies using multi-mycotoxin monitoring, adaptation of farming practice (varietal change, rotation, planting and harvest timing modulation), upgraded storage conditions, and the development of predictive models from climatic data and fungal ecophysiology (Perrone et al., 2020; Casu et al., 2024). During an era of rapid climate change, the resilience of the agri-food system will depend directly on the ability to forecast these changes and adopt pre-emptive measures towards minimizing exposure to mycotoxins (Bunny et al., 2024).

## 6 Mycotoxins and crop health

### 6.1 Effects of mycotoxins on plant physiology and resistance mechanisms

Mycotoxins produced by phytopathogenic fungi are not only food contaminants: they act as true virulence factors, altering plant physiology and compromising their defenses.

Deoxynivalenol (DON), the emblematic toxin of *Fusarium graminearum*, inhibits protein synthesis by binding to ribosomes and causes an accumulation of reactive oxygen species (ROS), leading to oxidative damage and sometimes programmed cell death (Audenaert et al., 2013). In wheat and barley, it reduces photosynthesis and root growth (Luo et al., 2023). Fumonisin B1 (FB1), produced by *F. verticillioides*, acts on sphingolipid metabolism by inhibiting ceramide synthase, causing membrane disruption and hypersensitivity to oxidative stress (Iqbal et al., 2021). As for aflatoxin B1, produced by *Aspergillus flavus*, it slows germination and reduces the biosynthesis of photosynthetic pigments, weakening seedlings (McLean et al., 1995). In general, many toxins, including trichothecenes, disrupt root development and limit nutrient absorption (Ansari et al., 2014).

**Figure 2:**
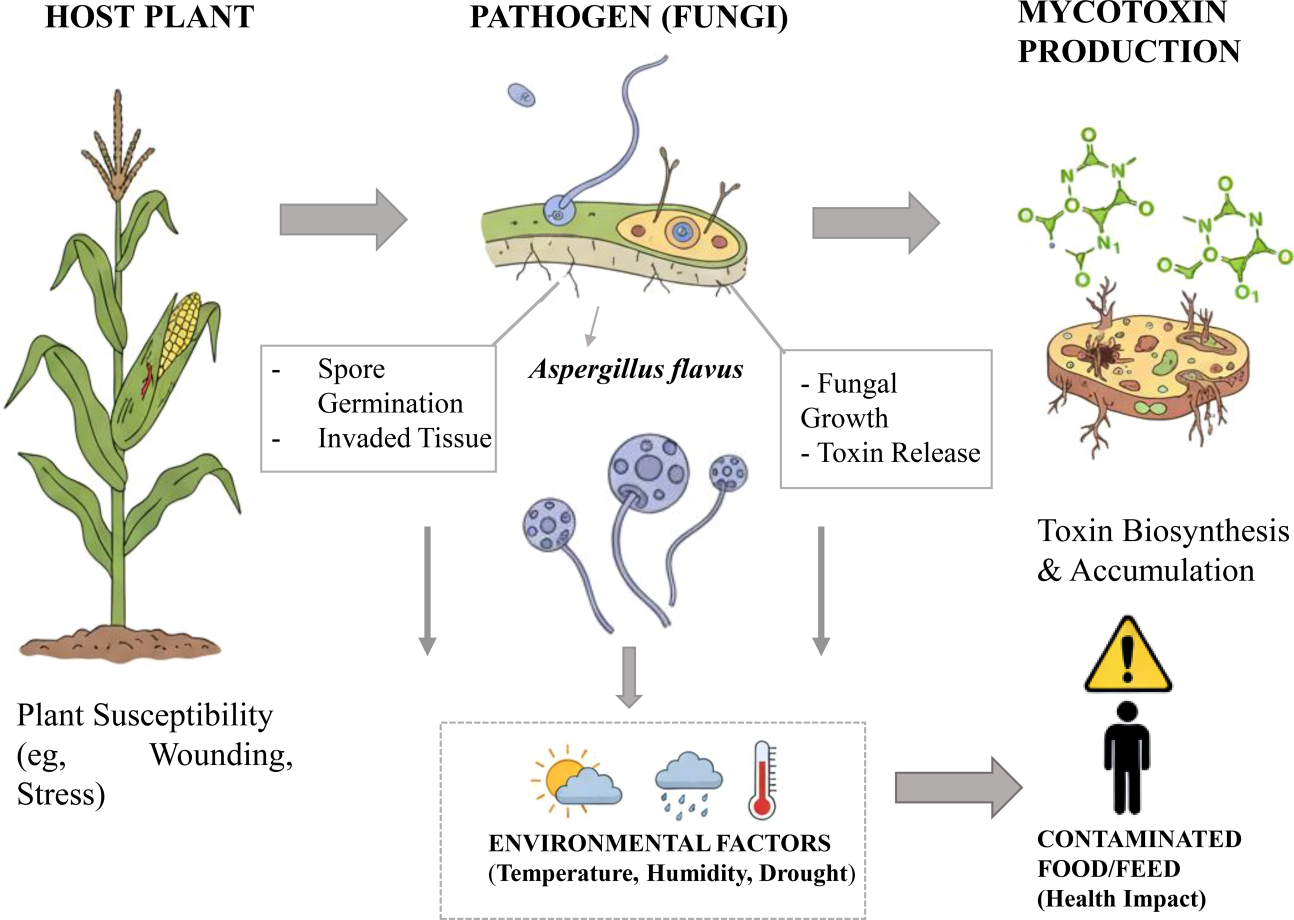
Plant-pathogen-mycotoxin interaction *Aspergillus*-Aflatoxin: opportunism and stress response.

Beyond their toxic effects, these molecules disrupt hormonal networks. DON interferes with jasmonic acid (JA) and salicylic acid (SA) signaling pathways, causing an unbalanced response that benefits *Fusarium* (Audenaert et al., 2013). FB1, meanwhile, triggers hypersensitive responses that are effective against biotrophs but exploited by necrotrophic fungi (Zeng et al., 2020). These observations show that mycotoxins function as true chemical effectors. However, plants have developed ways to neutralize these toxins. One of the best known is enzymatic conjugation: certain cereals convert DON into DON-3-glucoside (D3G), a less toxic molecule, using UDP-glucosyltransferases (Nagl et al., 2012; Berthiller et al., 2013). Similarly, detoxification reactions such as acetylation or hydroxylation reduce the reactivity of several mycotoxins (Li et al., 2020). At the same time, plants stimulate the production of phenolic metabolites and flavonoids, which are capable of both limiting oxidative damage and directly inhibiting toxin biosynthesis in fungi (Ismaiel and Papenbrock, 2015) (**Figure 2**).

In summary, mycotoxins profoundly disrupt plant physiology, from translation to photosynthesis, while manipulating hormonal defenses. Nevertheless, plants have enzymatic and metabolic strategies to limit these effects, providing a promising basis for the development of more resistant varieties.

### 6.2 Influence on microbiome shifts, root development, and defense signaling

Mycotoxins not only disrupt plant cell physiology; they also influence plant microbiome, root architecture, and defense signaling dynamics. These indirect effects shape plant–pathogen interactions and largely determine host resilience.

The presence of mycotoxins and associated pathogens can reshape the composition of the root and endophytic microbiome. For example, Zhu et al. (2023) showed that an endophytic fungus could reduce mycotoxin production by *Fusarium proliferatum* while altering the microbial composition of rice spikelets, favoring protective taxa such as *Pseudomonas* (Zhu et al., 2023). Similarly, a study on common beans revealed that infection by *Fusarium oxysporum* strongly alters the root microbiome, enriching certain bacteria such as *Flavobacterium* and *Pseudomonas* that may play a role in varietal resistance (Mendes et al., 2023). Finally, recent work on soybeans has confirmed that natural *Fusarium* infections are accompanied by major metagenomic and metabolomic changes, affecting the balance between beneficial and pathogenic microbes in the rhizosphere (van Bentum et al., 2025).

Roots are particularly vulnerable to the phytotoxic effects of mycotoxins. Trichothecenes, such as DON, limit primary root elongation and disrupt meristems, leading to reduced water and nutrient uptake (Ansari et al., 2014; Luo et al., 2023). FB1 interferes with root sphingolipids, altering membrane integrity and causing localized necrosis (Iqbal et al., 2021). These disturbances in the root system indirectly influence the overall health of the plant and its ability to interact with soil microbial communities. Recent work shows that, under biotic and abiotic stress, plants modify the quantity and composition of their root exudates, leading to rearrangements of the associated microbiome. In particular, altered exudate profiles can reduce the recruitment of beneficial microorganisms and increase the host’s vulnerability to pathogens (Sasse et al., 2018; Korenblum et al., 2020).

**Figure 3:**
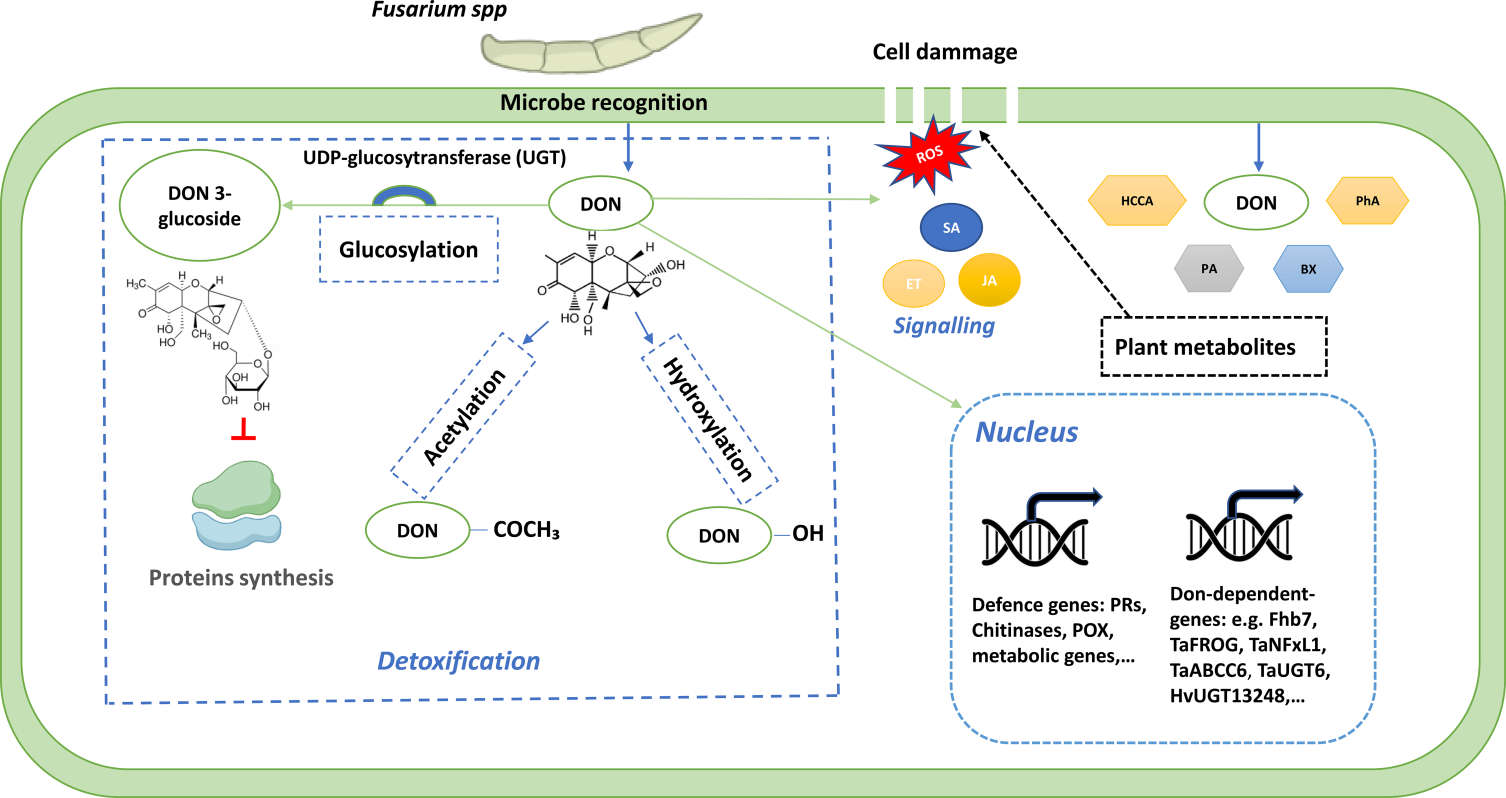
The cellular roles of mycotoxin deoxynivalenol (DON) in *Fusarium*–wheat interaction. Plant genes and pathways that are responsive to DON or influence the DON response and that are involved in *Fusarium* spp. disease resistance and susceptibility are illustrated. Detoxification of DON by UGTs is one of the main cellular mechanisms to counteract the deleterious effects of DON, followed by acetylation, and hydroxylation. DON induces the production of ROS and several plant defence metabolites that can influence plant cell wall composition and modulate *Fusarium* mycotoxin production. Abbreviations: BX, benzoxazinoid; DON, deoxynivalenol; ET, ethylene; HCCA, hydroxycinnamic acid amide, JA, jasmonic acid; PA, polyamine; PhA, phenolic acid; POX, peroxidase; PRs, pathogenesis-related; ROS, reactive oxygen species; SA, salicylic acid.

Mycotoxins also affect the signaling cascades involved in defense (**Figure 3**). DON, in particular, activates ROS accumulation and triggers the expression of genes linked to the jasmonic acid (JA) and salicylic acid (SA) pathways, but in an uncoordinated manner, creating a partially effective response (Nagl et al., 2012). FB1 triggers hypersensitive (HR) responses by activating sphingolipids and calcium signaling, which is unfavorable against necrotrophic fungi (Berthiller et al., 2013). These manipulations allow pathogens to bypass defenses and promote their progression into host tissues.

At the ecosystem level, these effects have significant implications. Disruption of the microbiome by mycotoxigenic fungi can reduce the resilience of agricultural soils to pathogens and limit beneficial long-term interactions (Nicolaisen et al., 2014; Pang et al., 2021). In addition, changes in defense signaling and root development reduce plant tolerance to abiotic stresses such as drought or salinity, exacerbating yield losses (van Bentum et al., 2025).

In summary, mycotoxins profoundly alter the balance between the plant and its biotic environment. By affecting the microbiome, they reduce beneficial microbial support; by disrupting roots, they compromise resource uptake; and by manipulating signaling pathways, they weaken the immune response. A better understanding of these interactions paves the way for integrated approaches combining microbiological management, genetic selection, and biotechnologies to limit the impact of these toxins.

## 7 Novel omics tools for mycotoxin and pathogen profiling

Omic technologies based on high-resolution mass spectrometry enable scientists to evaluate the three potential categories of existing mycotoxins, namely unmodified (native) forms of mycotoxins generated by different fungi (e.g., AF, OTA, ZEN, FB, PAT, and DON), followed by matrix-associated mycotoxins such as those non-covalently attached to peptides and polysaccharides, and metabolically modified (hidden or disguised) mycotoxins generated by fungi, bacteria, and cultures.

### 7.1 Metabolomic approach

Metabolites represent all low-mass molecular compounds, while the metabolic profile corresponds to the distribution of molecules (MW ≤ 1,000 Da) synthesized by living cells during their biochemical processes. These metabolites provide direct insight into the physiological functions of the cell (Zhang et al., 2012; Eshelli et al., 2015). Despite rapid advances in analytical technologies, our understanding of molecular communication between pathogenic fungi and their host species remains incomplete. Metabolomic analysis has emerged as an innovative tool for better understanding fungal biosynthesis mechanisms (Smedsgaard and Nielsen, 2005). Metabolites thus serve as indicators of cellular activity (Iriti and Faoro, 2009). This approach allows the quantification of functional phenotypes or biochemical signatures of disturbances in samples taken before or during the colonization of crops by mycotoxigenic fungi (Bhatnagar et al., 2008; Bergholz et al., 2014; Rychlik et al., 2017).

According to existing published research, biological fluids such as urine, serum, blood, plasma, and milk are the most frequently studied biofluids for targeted and untargeted metabolomic profiling associated with mycotoxin contamination (Castaldo et al., 2019; Owolabi et al., 2024).

Targeted metabolomics is generally used when researchers seek to quantify a defined set of metabolites to test a specific hypothesis. For example (Eshelli et al., 2015), applied targeted metabolomics to study the biological degradation of aflatoxin B1, or to detect various ranges of mycotoxin contamination in animal feed, food, and agricultural products (Arce-l et al., 2020; Rocchetti et al., 2021), whose analysis was based on UHPLC coupled with Orbitrap-HRMS. In contrast, untargeted metabolomics aims to detect as many metabolites as possible without prior selection, relying heavily on international databases such as METLIN, ChemSpider, and PubChem to facilitate compound identification (Alves et al., 2025).

In the context of mycotoxin research, it has been demonstrated that fungal metabolomes evolve during the growth and development of fungi on host plants (Falade et al., 2018), as well as in response to interactions with other microorganisms or environmental fluctuations (Powers and Riekeberg, 2017; Saldan et al., 2018). A recent review by Falade et al. highlighted the correlation between metabolites produced during fungal growth on corn (e.g., trehalose, mannitol, sorbitol) and the accumulation of aflatoxins, emphasizing their importance in toxin biosynthesis (Bhatnagar et al., 2008).

Beyond analytical profiling, metabolomics has provided direct insights into plant–fungal interactions and pathogenicity mechanisms. For instance, metabolomic analyses have revealed that infection of maize and peanut by Aspergillus flavus is accompanied by coordinated changes in host carbohydrate metabolism and antioxidant compounds, which are closely linked to aflatoxin biosynthesis. Similarly, studies on wheat–Fusarium interactions have shown that the accumulation of specific sugars, polyols, and phenolic compounds during infection correlates with fungal virulence and mycotoxin production, highlighting metabolomics as a powerful tool for elucidating host susceptibility and fungal pathogenic strategies (Bhatnagar et al., 2008; Falade et al., 2018).

#### 7.1.1 Analytical techniques in mycotoxin metabolomics

Early studies focused on the quantification of individual mycotoxins. Thin-layer chromatography (TLC) was originally used by Scott et al. during 1970, effectively identifying 18 fungal toxins, such as aflatoxins variants B2, B1, G2, G1, and ochratoxin type A. These were separated using with solvent application for systems such as ethyl acetate - formic acid or benzene – toluene-methanol–acetic acid, subsequently treated with p-anisaldehyde visualization reactant. Aflatoxin B1 and ochratoxin A were visualized under UV light as green and blue, fluorescent spots, respectively (Rieswijk et al., 2016).

Since then, a wide range of chromatographic techniques have been employed, including high-performance liquid chromatography (HPLC) and gas chromatography (GC), often in combination with mass spectrometry (MS) to form so-called hyphenated techniques. Examples include GC–MS, GC–MS/MS, LC–MS, LC–MS/MS, and LC–NMR–MS, which remain the most widely applied analytical tools for mycotoxin detection (Iriti and Faoro, 2009; Oueslati et al., 2012; Zhang et al., 2012; Adekoya et al., 2018). The change starting with high-performance liquid chromatography into ultra-high-performance liquid chromatography (UHPLC) has significantly enhanced throughput, enabling the detection of additional metabolites within shorter processing times.

Ultra-high-performance liquid chromatography linked with four-pole mass filter -orbital ion trap mass spectrometry has been applied to identify 26 frequent fungal toxins such as aflatoxins B2, G2, B1, G1, and ochratoxins A and B in grains and nuts such as almonds, wheat, corn, rice, peanuts, also pistachios. This highlights the capacity pertaining to hybrid mass spectrometry for food safety monitoring (Zhang et al., 2014). Rubert et al. applied ultra-high-performance liquid chromatography–quadrupole time-of-flight to investigate plant– disease interaction, generating metabolic fingerprints that facilitated the innovation relating to bio-surveillance tools the purpose of early mycotoxin detection in wheat (Rubert et al., 2017). Similarly (Oplatowska-Stachowiak et al., 2015), applied ultra-high-performance liquid chromatography–tandem mass spectrometry for quantifying mycotoxins in dried grain.

Among these, LC–MS/MS is currently the maximum widely used method for targeted the evaluation of recognized mycotoxins. In this approach, analytes are fragmented via collision-induced dissociation, and the resulting ions are detected. Triple quadrupole (qQq) instruments operating in single reaction monitoring (SRM) mode are especially favored for their high sensitivity and selectivity, enabling quantification of mycotoxins at the microgram scale (Malachová et al., 2018). However, LC–MS/MS is limited to detecting predefined metabolites and cannot identify transformed or modified toxins unless they are already characterized (Hopfgartner et al., 2004).

In contrast, LC–HRMS (high-resolution MS) has emerged as a more versatile approach due to its superior resolution and ability to record accurate masses for all ions generated, independent of fragmentation patterns. This allows both targeted and untargeted analyses within a single workflow (Hird et al., 2014; Malachová et al., 2018). Time-of-flight (TOF) and Orbitrap-based HRMS instruments are now standard tools for untargeted metabolomics due to their sensitivity, mass accuracy, and capability to tentatively identify both known and novel metabolites (Eshelli et al., 2015; Righetti et al., 2016).

Recent efforts have also incorporated chemometric analysis, a powerful mathematical and statistical approach that extracts relevant chemical information from complex datasets. These strategies have enabled multi-mycotoxin analysis in cereal samples, often without extensive clean-up steps (Aretz and Meierhofer, 2016). Such developments emphasize the importance of advanced HRMS platforms in expanding the scope of mycotoxin metabolomics.

### 7.2 Transcriptomics approach

Transcriptomics is one of the most recently established disciplines to emerge born of the genomics revolution (Dong and Chen, 2013). Following the finalization of genomic research, scientific focus shifted toward exploring the subsequent steps of genomic transcriptional activity and cellular-level function. Since the hereditary information encoded in DNA it’s not able to be directly translated within polypeptides, an intermediate process transcription is required, whereby DNA is copied into RNA. This represents a crucial control stage in transcriptional activity (Scheper et al., 2007).

Transcriptomics, therefore, serves as the investigation of the full complement of RNA transcripts produced by the genetic material during environments in a specific cell or tissue type (Garcia-Cela et al., 2018; Reddy et al., 2018). Transcript evaluation enables scientists to investigate genome translation at the transcriptional level, thereby providing insights into gene structure (Pedrotty et al., 2012). For example, it can determine whether a sequence encodes a functional product or an intermediate later translated into protein, as well as track increases or decreases in protein production (control of gene expression), post-translational, functions of gene products, modifications, and evolutionary adaptations of physiological pathways (Tian et al., 2020).

Genes are thus regulated with expressed differently depending on biological and physiological conditions, resulting in the synthesis of distinct proteins. For instance, transcripts derived from mycotoxin-infected plant cells differ from those in non-infected plant cells, highlighting their role in communication and biomolecular pathways (Zumaquero et al., 2019). Combined with innovative assessment approaches, transcriptomics plays a key function in elucidating highly organized physiological networks and contributes to the development of recent molecular indicators (Ling et al., 2017). Consequently, it holds strong capacity toward initial phase detection and the identification of effective therapeutic or agricultural strategies (Sayanthooran et al., 2017). Moreover, transcriptome analysis can further uncover regulatory networks of biological processes, ultimately supporting crop improvement initiatives (Pedrotty et al., 2012).

Transcriptomic approaches have further enabled the identification of key genes involved in fungal pathogenicity and host defense responses during infection. For example, RNA-seq analyses of wheat infected with Fusarium graminearum have revealed the upregulation of fungal genes involved in deoxynivalenol biosynthesis alongside the activation of plant defense-related genes associated with jasmonic acid, salicylic acid, and phenylpropanoid pathways. Similar transcriptomic studies in maize–Aspergillus interactions have demonstrated that host resistance is associated with the differential expression of genes involved in oxidative stress regulation and cell wall reinforcement, thereby providing mechanistic insights into disease development and resistance (Garcia-Cela et al., 2018; Reddy et al., 2018).

### 7.3 Artificial intelligence

Artificial intelligence (AI) techniques have proven to be highly effective in detecting mycotoxins and mycotoxigenic fungi across a wide range of food products, offering advantages such as reliability, cost-effectiveness, rapid processing, and the ability to handle uncertainty (Chowdhury and Sadek, 2012; Onkol, 2020). In peanuts, aflatoxin B1 (AFB1) was identified using approaches that combined genetic algorithms, backpropagation neural networks, and support vector regression, with sparrow search optimization achieving the best predictive performance (R = 0.91); other models such as SVM, ANN, and ANFIS also demonstrated strong efficiency, with ANN-based detection under LED light reaching 99.7% accuracy (Ziyaee et al., 2021). Multispectral imaging coupled with ML algorithms like SVM, RF, MLP, and LDA achieved 90–100% accuracy for fungal classification (Sudki et al., 2023), while SVM and DCNN applied to optical coherence tomography identified moldy peanuts with 85–96% accuracy; transformer models further improved detection accuracy (Manhando et al., 2021). In wheat, CNN and DCNN models efficiently detected Fusarium head blight (FHB) and AFB1, reaching up to 99% accuracy, with fusion CNN architectures achieving perfect scores for accuracy, precision, recall, and F1 (Deng et al., 2023); e-nose systems such as the AIR PEN 3 also proved effective for rapid detection of deoxynivalenol (Camardo Leggieri et al., 2021). Similar approaches were used in rice, where DBN, CNN, SVM, and BPNN models classified mold contamination with up to 100% accuracy, supported by e-nose validation (Gu et al., 2019). In barley, hyperspectral imaging combined with CNN, SVM, RF, KNN, and SGD reached 89.81% accuracy in classifying deoxynivalenol levels (Fan et al., 2023), while in maize, CNN with hyperspectral imaging and sparse auto-encoders achieved 99.47% recognition, and AI models integrating meteorological and satellite data effectively predicted aflatoxin and fumonisin outbreaks (73% and 85%, respectively) (Yang et al., 2022); additional methods such as Raman spectroscopy with LDA or SVM, RF with NIR data (Ghilardelli et al., 2022), and BPNN-DL for zearalenone (Liu et al., 2022b). further enhanced prediction accuracy. Beyond cereals, AI applications extended to apples, where e-nose and PLS models predicted patulin (Wang et al., 2017), and smartphone-based aptasensors selectively detected multiple mycotoxins (Erdem and Senturk, 2024); in milk, nano-biosensors combined with spectral detection improved antibiotic residue monitoring (Gutiérrez et al., 2020). Deep learning also achieved high accuracies in cocoa beans (GoogLeNet-CNN, 96.42%) (Gutiérrez et al., 2020), coffee (NIR spectra), edible oils (CNN/RNN, 100% for qualitative identification) (Jiang et al., 2023), almonds (fluorescence imaging with DNNs, 84.7–93.0%) (Bertani et al., 2024), and figs (UV-based transfer learning, 98.57%) (Kılıç and İnner, 2022). Moreover, ML models predicted *Fusarium* growth and toxin production under varying conditions (Tarazona et al., 2021). Collectively, these studies highlight AI, including ML, DL, CNNs, transformers, and biosensor integration, as reliable, rapid, and non-destructive tools for safeguarding food safety through accurate detection of mycotoxins and fungal contamination.

Collectively, these omics-based studies demonstrate that metabolomics and transcriptomics not only facilitate mycotoxin detection but also significantly advance our understanding of fungal pathogenesis and host–pathogen interactions at the molecular level.

### 7.4 Omics- and AI-enabled approaches across the crop–food continuum

Omics and artificial intelligence (AI) are increasingly used to manage mycotoxigenic fungi and mycotoxins through an integrated “crop-to-consumption” perspective, beginning at crop production where plant–fungal interactions and environmental stress shape infection dynamics. In the field, predictive modeling and machine learning can combine meteorological variables, soil data, and remote sensing indicators to anticipate contamination risk in major plant products (e.g., maize, wheat, peanuts) and support timely agronomic decisions (e.g., harvest timing, irrigation management, targeted scouting). For example, machine-learning models have been developed to predict aflatoxin and fumonisin contamination in maize using historical contamination records and environmental predictors, demonstrating the potential of data-driven early warning systems for preharvest risk management (Castano-Duque et al., 2022).

Beyond prediction, omics platforms (notably LC–HRMS-based workflows) strengthen field-to-lab surveillance by enabling multi-analyte detection of regulated, modified, and emerging mycotoxins in crop matrices. Such approaches are valuable not only for food safety monitoring but also for understanding how fungal metabolism and toxin profiles vary with host status and environmental conditions, thereby providing mechanistic insight into plant–fungal interactions (Eshelli et al., 2018).

After harvest, post-harvest handling and storage remain decisive control points because small increases in moisture or temperature can rapidly shift fungal activity and mycotoxin accumulation in stored grains and nuts. Here, omics-based multi-screening methods support comprehensive profiling of co-occurring toxins, while AI-assisted tools (including spectroscopy coupled with machine learning) offer rapid screening approaches that can help prioritize confirmatory analyses and guide lot segregation (Lapris et al., 2024).

## 8 Integrated disease and mycotoxin management

### 8.1 Breeding for resistance against toxigenic fungi

The option of selecting crop varieties resistant to major toxic phytopathogenic fungi, such as *Fusarium graminearum* and *F. verticillioides*, is a crucial approach to decrease both the incidence of fungal diseases and the accumulation of mycotoxins (such as deoxynivalenol and fumonisin) in seeds (Mesterhazy, 2024). Studies have revealed genetic variation within maize populations regarding fumonisin production and the extent of Fusarium ear rot (FER), demonstrating strong genetic correlations (r ≈ 0.87–0.96) and moderate to high heritability. This paves the way for indirect phenotypic selection based on visual assessment of ear rot (Santiago et al., 2020). Traditional plant breeding methods, such as pure-line selection and biparental crosses, have led to improvements in resistance to fungal pathogens (Maschietto et al., 2017). identified overlapping quantitative trait loci (QTLs) for fumonisin B_1_ concentration and FER severity on linkage groups 1, 2, 3, 6, 7, and 9 of an F_3_ maize population, facilitating the selection of genotypes with dual resistance. Similar inoculation trials conducted on groups of commercial hybrids showed that only 10–15% of them exhibited reduced fungal infection and toxin accumulation. This underscores the need to evaluate resistance traits under representative environmental conditions (Mesterhazy et al., 2022). Meta-analyses have been crucial for refining QTL data (Akohoue and Miedaner, 2022). performed a reanalysis of 224 QTLs related to Gibberella ear rot (GER), FER, deoxynivalenol, and fumonisin accumulation, and silk and grain resistance traits (**Table 5**). They identified 40 meta-QTLs (MQTLs), 28 of which are shared by both GER and FER characters. These MQTLs, including ZmMQTL9.4, ZmMQTL9.2, and ZmMQTL2.2, comprise candidate genes such as flavonoid O-methyltransferase 2 (fomt2) and terpene synthase (tps21), which are promising targets for breeding programs aiming for broad-spectrum resistance to mycotoxins and ear rot. Despite the advances achieved through marker-assisted selection (MAS) in plant genetics, its effectiveness is often constrained when traits are controlled by many loci with small individual effects. In contrast, genomic selection (GS) integrates genome-wide marker information to capture the cumulative effects of numerous minor loci, making it a promising alternative for improving complex, polygenic traits. According to a study by (Asiedu and Miedaner, 2025) on selection tools, GS has significant potential for combating *Fusarium* stalk rot (FSR). However, its current application is limited and could be improved by combining it with “omics” validation techniques. Research in genomic prediction and genome-wide association studies (GWAS), such as that conducted by (Liu et al., 2021) on 874 tropical maize lines, also demonstrates the effectiveness of GS in identifying polygenic effects. In addition to traditional selection approaches based on quantitative genetics, genome editing technologies and contemporary functional genomics provide extremely precise techniques for genetic modification (Liu et al., 2022a). generated null mutants of the ZmFER1 susceptibility gene using CRISPR/Cas9 technology. These mutations resulted in maize varieties with enhanced resistance to *Fusarium verticillioides*, while maintaining their agronomic performance. Furthermore (Ottaviani et al., 2025), demonstrated that inactivation of the transcription factor ZmWRKY125, achieved through CRISPR/Cas9 technology, enhances resistance by regulating phytohormone signaling, neutralizing reactive oxygen species (ROS), and modulating secondary metabolism. In addition to these methods (Yang et al., 2025), performed a functional characterization of the ZmPR5 protein, which is closely linked to the pathogenesis process. They demonstrated that its overexpression, as well as the use of a mutant obtained through EMS mutagenesis, induced increased activation of defense mechanisms through membrane stabilization and antioxidant effects.

**Table 5:** Integrated strategies to reduce mycotoxin accumulation and fungal infections in staple crops under climate change.

Crop/pathogen	Representative resistant variety or case study	Key breeding or biotechnological strategy	Main mechanisms (target traits)	References
Wheat/*Fusarium graminearum*	‘Sumai 3’-derived lines with improved Fusarium Head Blight resistance	Introgression of Fhb1 and novel QTL; genome editing of deoxynivalenol-detoxification pathways	Enhanced cell-wall reinforcement, increased UDP-glucosyltransferase activity, and reduced deoxynivalenol accumulation	(Zhang et al., 2024b; Luo et al., 2025)
Rice/*Fusarium fujikuroi* (Bakanae disease)	IRRI breeding lines tolerant to Bakanae	MAS for resistance QTL; CRISPR editing of susceptibility genes	Enhanced lignification, suppression of pathogen invasion	(Hur et al., 2015; Boubakri, 2023)
Maize/*Fusarium verticillioides*	CIMMYT hybrids under African climate-change scenarios	Genomic prediction for fumonisin resistance; pyramiding QTL for kernel integrity and detoxification enzymes	Tight husk coverage, upregulation of fumonisin-detoxifying enzymes	(Duvick, 2001; Nyandi, 2025)
Maize/*Aspergillus flavus (aflatoxin)*	Inbred line Mp313E (USA) with combined resistance to *A. flavus* and reduced aflatoxin	Marker-assisted introgression of QTL for ear rot resistance; genomic selection combined with CRISPR/Cas9 editing of antifungal protein genes	Enhanced pericarp thickness, increased antifungal protein expression, and reduced aflatoxin biosynthesis	(Brown et al., 2013; Karim et al., 2025a)
Barley/*Fusarium* spp.	European malting barley with reduced deoxynivalenol	MAS plus genomic selection for Fusarium Head Blight; deployment of detoxifying alleles	Early flowering to escape infection, improved kernel morphology	(Buerstmayr et al., 2020; Ghazy et al., 2024)
Sorghum/*Claviceps africana* (Ergot)	Sorghum hybrids in sub-Saharan Africa	Genomic prediction for floral traits; CRISPR editing of stigma exsertion	A shorter anthesis-stigma interval reduces ergot infection	(Asiedu and Miedaner, 2025; Kebede et al., 2025)
Groundnut/*Aspergillus flavus*	ICRISAT lines with combined drought and aflatoxin resistance	Genomic selection combined with wild Arachis introgression; CRISPR-mediated alteration of seed lipid profile	Reduced seed micro-cracks, increased antioxidant content, and limited aflatoxin accumulation	(Khadgi et al., 2025)

Modern breeding for resistance to toxigenic fungi increasingly relies on an integrated strategy that combines several complementary approaches (Ivanov et al., 2021). Traditional QTL mapping and marker-assisted selection (MAS) are employed to identify genes with major effects (Buerstmayr et al., 2020), while meta-QTL analysis helps pinpoint stable genomic regions conferring resistance across multiple traits (Venske et al., 2019). Genomic selection is used to capture the cumulative contribution of minor alleles (Poland and Rutkoski, 2016), and CRISPR-based genome editing enables the precise enhancement of resistance genes (Schenke and Cai, 2020). In addition, functional validation through ‘omics’ technologies and molecular biology techniques provides crucial insights into candidate gene activity (Jin et al., 2021). Together, these approaches form a robust framework for developing crop cultivars with improved resilience to climate change, capable of reducing both fungal infections and mycotoxin accumulation (Casu et al., 2024).

### 8.2 Endophytes and biocontrol agents: dual protection against toxins and pathogens

The use of biocontrol agents (BAs) and endophytic microorganisms appears to be a promising approach for fighting pathogenic fungi and diminishing mycotoxin levels in agroecosystems, mainly under challenging environmental conditions. Species of the genus *Trichoderma* stand out among these BAs due to their versatile antagonistic mechanisms (Farhaoui et al., 2023b, Farhaoui et al., 2025). For example, *T. harzianum* K179 has demonstrated a strong antagonistic effect *in vitro* against *Aspergillus flavus* and *F. graminearum*, with inhibition rates reaching 55% and 69%, respectively. *In vivo* field trials using seed treatments, including seed coating and seed soaking, demonstrated a significant reduction in the severity of fungal infections and an increase in the number of healthy maize ears compared to untreated controls or treatments using chemical fungicides (Mitrović et al., 2025). Additionally, investigations on mycotoxin concentrations have shown that the use of *T. harzianum* K179 resulted in significantly lower levels of zearalenone, aflatoxins, and fumonisins compared to samples treated with fungicides or untreated control samples, even under extreme weather conditions (Mitrović et al., 2025). Several studies have shown that *Trichoderma* spp. are known for their ability to eliminate pathogenic fungi through mechanisms such as mycoparasitism, antibiosis, competition, and the induction of systemic resistance. Furthermore, they stimulate plant growth and enhance nutrient uptake (Farhaoui et al., 2022, Farhaoui et al., 2023a). It has also been demonstrated that certain *Trichoderma* strains are capable of enzymatically transforming *Fusarium* toxins into less less toxic metabolites, including the biotransformation of deoxynivalenol (DON) into epimerized or conjugated forms (e.g., 3-epi-DON and DON-3-glucoside), thereby contributing to both direct detoxification and indirect mitigation of toxin-associated damage (Yao et al., 2023).

Endophytic microorganisms, such as bacteria and fungi, offer an additional means of biological control. By colonizing the internal tissues of plants, they modulate the plant’s defense mechanisms and can even degrade mycotoxins. A study combining *in vivo* and *in vitro* tests demonstrated that endophytic bacterial strains (EPC2, EPR2, EPL3) isolated from maize inhibited colonization by *Aspergillus parasiticus* and reduced aflatoxin levels by approximately 95%, from 445 ppb in the untreated control group to about 23 ppb in the group treated with these endophytic strains (Sivakaame et al., 2025). Fungal endophytes, such as *Trichoderma* and *Penicillium*, act through interspecific competition, releasing antifungal molecules and stimulating systemic resistance in the plant, which reduces contamination by several mycotoxins while strengthening the resilience of the host plant (Sivakaame et al., 2025). A meta-analysis revealed the existence of a wide variety of bacterial (such as *Bacillus licheniformis* CFR1) and fungal strains capable of eliminating toxic fungal species and inactivating various mycotoxins. For example, *B. licheniformis* CFR1 reduced the concentration of aflatoxin B1 by approximately 95% while also decreasing the mutagenic potential of this toxin (Raksha Rao et al., 2017; Helal et al., 2025). Numerous studies on biological control strategies also highlight the use of edible fungi, lactic acid bacteria, and enzymatic methods for neutralizing mycotoxins (Ali et al., 2023).

BAs, particularly species of *Trichoderma* and endophytic microorganisms, employ various mechanisms (such as eliminating plant pathogens, degrading toxins, and stimulating the host plant’s defenses) that make them essential tools for the integrated management of plant diseases and mycotoxins. Field trial results provide a solid foundation, while improved strains, developed through advances in biotechnology (designed for enhanced detoxification), represent promising potential for the future.

### 8.3 Post-harvest procedures: storage conditions, detoxification processes, and innovative packaging

Post-harvest management is crucial to reduce the danger of mycotoxins, especially in the context of global warming, which favors the expansion of fungi. Improving storage conditions, especially temperature and humidity management, is essential for the effectiveness of a post-harvest method. According to several studies, the acceleration of fungal growth, particularly *Penicillium* and *Aspergillus* species, occurs when grain moisture content exceeds 13–13.5%, reaching its peak between 13.5 and 18–20% (Wyllie, 1990). It is therefore crucial to keep humidity below this level and to carry out rapid drying to avoid the production of mycotoxins (Farhaoui et al., 2023b). Based on these preventive actions, physical sorting and decontamination have an additional role. For example, mechanical cleaning of maize kernels resulted in an 83.6% reduction in deoxynivalenol without altering the surface (Bakari, 2023). Similarly, laser sorting of peanuts effectively removed aflatoxin-contaminated kernels at speeds ranging from 2.4 to 4 tons per hour, while minimizing yield losses (Wyllie, 1990). Chemical and non-thermal treatment methods allow for more efficient detoxification. According to (Zhao et al., 2023), ozone exposure—within half an hour—was observed to remove 85–100% of aflatoxins and patulin from chickpea seeds, while nitrogen-rich environments (>94%) resulted in comparable reduction of mycotoxins in milling matrices. Non-thermal technologies such as pulsed light, cold plasma, and the use of nanomaterials (e.g., graphene/ZnO, TiO_2_) show interesting potential. However, their disadvantages include generally limited surface effectiveness, high costs, and potential threats to food quality (Wei et al., 2023). The biological detoxification process represents a sustainable and delicate strategy, particularly thanks to lactic acid bacteria that have the power to bind and metabolize mycotoxins. Studies demonstrate that lactic acid bacteria are capable of effectively detoxifying major mycotoxins on a large scale (Petrova et al., 2022), while a pilot implementation of *Lactobacillus reuteri* R29 resulted in an 83% reduction in deoxynivalenol in malted barley (Oliveira et al., 2015). In addition, the use of microbial biofilms—which are still in their early stages—led to an aflatoxin M1 fixation of approximately 60% (Muhialdin et al., 2020). Finally, new developments in packaging add a dimension of active protection. Active and improved packaging systems, such as humidity controllers and antimicrobial protections, can inhibit fungal growth during storage and transport (Fumagalli et al., 2021). According to (Magan and Aldred, 2007), controlled atmosphere storage, particularly with CO_2_ concentrations above 75%, effectively eliminates mycotoxin-producing molds in semi-moist grains.

## 9 Conclusion and future directions

The connected worldwide issue of how climate change impacts mycotoxins include places, farming practices, and the economic resources of communities. The natural roles, biological processes, and the ability to compete of fungi that make mycotoxins are greatly changed by increasing world temperatures, different amounts of rain, and changing levels of dampness. Some types from warm areas, like *Aspergillus flavus* (which makes aflatoxins), are moving to cooler areas, while others, like *Fusarium* spp., which make fumonisins, deoxynivalenol (DON), and zearalenone (ZEN), are either finding new places that suit the weather or being pushed out by competitors that can handle hotter weather. These changes are not only about location; they also change when sicknesses occur, the different types of toxins made, and the existence of several mycotoxins in one item, which makes it harder to measure and deal with dangers. Furthermore, fungal colonization and mycotoxin production increase as a result of weakened plant immune defenses due to climate-induced stressors on crops. Fungal metabolism and mycotoxin production are accelerated by the interaction of elevated CO_2_ levels with temperature and water availability. These conditions have a direct negative impact on global trade and food security due to the transfer of contaminated foodstuffs. Most alarming is the novel discovery of “masked” or modified mycotoxins, including conjugate types that have arisen during plant–pathogen interactions. Less toxic when they initially penetrate the organism, these molecules can be reactivated by digestion to their original form, evading standard digestion methods and posing an unsuspected degree of risk of contamination. Innovative predictive models and local monitoring techniques should be combined in future studies to forecast mycotoxin profile trends under anticipated climate regimes. Future research should integrate local monitoring with predictive models to analyze mycotoxin trends under climate change, necessitating biocontrol, crop rotation, precision irrigation, and resistant varieties. To reduce postharvest contamination and stop the development of mycotoxins, new storage solutions are also required. Integration of plant phenology, meteorological data, and fungal genetics is necessary for effective early warning systems. Regulatory frameworks must modernize safety standards and analytical techniques to counteract co-contamination and new poisons. Food safety systems can be strengthened against mycotoxin risks by promoting transdisciplinary collaboration across different sectors and fortifying international research networks.
